# The binding site for neohesperidin dihydrochalcone at the human sweet taste receptor

**DOI:** 10.1186/1472-6807-7-66

**Published:** 2007-10-12

**Authors:** Marcel Winnig, Bernd Bufe, Nicole A Kratochwil, Jay P Slack, Wolfgang Meyerhof

**Affiliations:** 1 German Institute of Human Nutrition Potsdam-Rehbruecke, Department of Molecular Genetics, Arthur-Scheunert Allee 114-116, 14558 Nuthetal, Germany; 2 Pharmaceuticals Division, F. Hoffmann-La Roche Ltd, Grenzacherstrasse 124, 4070 Basel, Switzerland; 3 Givaudan Flavors Corporation, 1199 Edison Drive, Cincinnati, 45216 Ohio, USA; 4 Axxam, San Raffaele Biomedical Science Park, via Olgettina 58, 20132 Milan, Italy; 5 Department of Physiology, University of Saarland School of Medicine, 66421 Homburg/Saar, Germany

## Abstract

**Background:**

Differences in sweet taste perception among species depend on structural variations of the sweet taste receptor. The commercially used isovanillyl sweetener neohesperidin dihydrochalcone activates the human but not the rat sweet receptor TAS1R2+TAS1R3. Analysis of interspecies combinations and chimeras of rat and human TAS1R2+TAS1R3 suggested that the heptahelical domain of human TAS1R3 is crucial for the activation of the sweet receptor by neohesperidin dihydrochalcone.

**Results:**

By mutational analysis combined with functional studies and molecular modeling we identified a set of different amino acid residues within the heptahelical domain of human TAS1R3 that forms the neohesperidin dihydrochalcone binding pocket. Sixteen amino acid residues in the transmembrane domains 2 to 7 and one in the extracellular loop 2 of hTAS1R3 influenced the receptor's response to neohesperidin dihydrochalcone. Some of these seventeen residues are also part of the binding sites for the sweetener cyclamate or the sweet taste inhibitor lactisole. In line with this observation, lactisole inhibited activation of the sweet receptor by neohesperidin dihydrochalcone and cyclamate competitively, whereas receptor activation by aspartame, a sweetener known to bind to the N-terminal domain of TAS1R2, was allosterically inhibited. Seven of the amino acid positions crucial for activation of hTAS1R2+hTAS1R3 by neohesperidin dihydrochalcone are thought to play a role in the binding of allosteric modulators of other class C GPCRs, further supporting our model of the neohesperidin dihydrochalcone pharmacophore.

**Conclusion:**

From our data we conclude that we identified the neohesperidin dihydrochalcone binding site at the human sweet taste receptor, which overlaps with those for the sweetener cyclamate and the sweet taste inhibitor lactisole. This readily delivers a molecular explanation of our finding that lactisole is a competitive inhibitor of the receptor activation by neohesperidin dihydrochalcone and cyclamate. Some of the amino acid positions crucial for activation of hTAS1R2+hTAS1R3 by neohesperidin dihydrochalcone are involved in the binding of allosteric modulators in other class C GPCRs, suggesting a general role of these amino acid positions in allosterism and pointing to a common architecture of the heptahelical domains of class C GPCRs.

## Background

Genetic, anatomical and functional studies provide compelling evidence that the vast majority of sweet taste perception is mediated by G-protein coupled receptors (GPCR) of the TAS1R-gene family, which comprises the members TAS1R1-3 [1-6]. TAS1Rs belong to the class C GPCRs and are distantly related to the calcium sensing receptor, metabotropic glutamate receptors, V2R pheromone receptors, and GABA_B _receptors [3]. In situ hybridization studies revealed that TAS1R3 is coexpressed with TAS1R1 or TAS1R2 in taste receptor cells [5,6]. This observation suggests that the functional receptor, like other class C GPCRs [7], may be a heteromer of two subunits. Indeed, functional assays revealed that the combination of TAS1R1+TAS1R3 is activated by umami tasting compounds, while TAS1R2+TAS1R3 responds to sweeteners [4,5,8].

The human sweet taste receptor is sensitive to the sweet proteins thaumatin, brazzein and monellin, the artificial sweeteners aspartame and cyclamate as well as to the sweet inhibitor lactisole whereas its rodent homolog is not [4]. This is in line with corresponding variations in sweet perception across species [9-11]. Studies with chimeric receptors revealed that replacement of the large N-terminal extracellular domain of rat Tas1r2 by its human counterpart created a receptor that responded to aspartame and neotame when coexpressed with hTAS1R3. Additional mutations in the N-terminal domain of human TAS1R2 impaired the activation of the sweet taste receptor by aspartame, thus suggesting that the N-terminal part of TAS1R2 is involved in the binding of these sweeteners [12,13]. Similar studies showed that amino acids in the cysteine-rich region of human TAS1R3 that connects the N-terminal extracellular domain to the segment containing the heptahelical domain determines the response to sweet proteins such as brazzein and monellin [12]. Conversely, replacement of the heptahelical domain of rat Tas1r3 by the corresponding part of the human receptor led to a chimera that responded to lactisole and cyclamate when coexpressed with rat Tas1r2 [13]. This suggests that the binding sites for cyclamate and lactisole are located in the heptahelical domain of hTAS1R3. Indeed, mutational analysis in combination with molecular modeling studies of the heptahelical domain of TAS1R3 revealed that the sweet inhibitor lactisole and the sweetener cyclamate have overlapping binding sites in the heptahelical domain of the human TAS1R3 subunit [14,15]. Recently, analysis of rat-human sweet taste receptor chimeras revealed that the heptahelical domain of hTAS1R3 is also crucial for the activation by the sweetener neohesperidin dihydrochalcone (NHDC) [16]. NHDC is added to various foods and beverages as a low caloric sweetener [17], but its use is limited by some unwanted sensory properties such as a delayed onset and a long lingering menthol-licorice like sweetness [18,19]. Thus, a detailed molecular understanding of the interactions of the sweet receptor with NHDC may contribute to the rational design of analogues with improved sensory properties. We therefore investigated the binding pocket of neohesperidin dihydrochalcone at the human sweet taste receptor.

## Results

### Analysis of receptor chimeras reveals that NHDC requires the human TAS1R3 heptahelical domain

**Figure 1:**
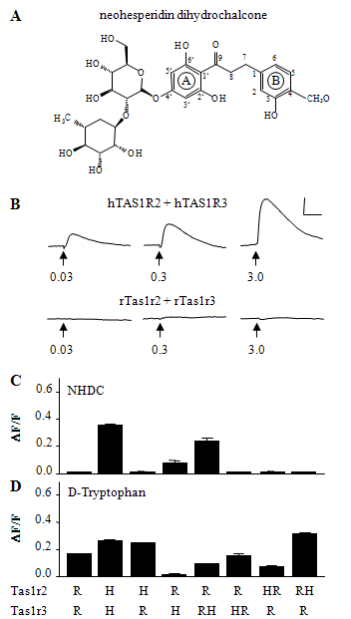
**Chemical structure and calcium responses elicited by neohesperidin dihydrochalcone**. (A) Structure of neohesperidin dihydrochalcone. Numbers denote carbon atom positions. (B) Representative calcium traces elicited upon stimulation with different concentrations of neohesperidin dihydrochalcone (NHDC) in HEK293T-G16Gust44 cells cotransfected with DNA for hTAS1R2/hTAS1R3. (C,D) Calcium responses of cells cotransfected with DNA for different rat and human *TAS1R *subunits or chimeras upon stimulation with 1 mM NHDC (C), or 30 mM D-Tryptophan (D). R, rat receptor subunit; H human receptor subunit; HR, receptor chimera comprising the N-terminal extra cellular domain of the human receptor subunit fused to the corresponding heptahelical domain of the rat; RH, comprising the N-terminal extracellular domain of the rat receptor subunit fused to the corresponding heptahelical domain of the human receptor.

The isovanillyl compound NHDC (Fig. 1A) activates the human but not the rodent sweet taste receptor (Fig. 1B). In order to elucidate the putative binding site for NHDC, we cotransfected mixtures of plasmid DNAs for the rat and human TAS1R2 and TAS1R3 receptor subunits in HEK293T cells stably expressing the chimeric G-protein G16Gust44 and measured cellular calcium responses by fluorometry following bath application of various sweet tasting compounds. We found that NHDC elicited calcium responses in cells transfected with human TAS1R3 and rat Tas1r2 cDNA, whereas it did not in cells transfected with the opposite combination (Fig. 1C). By replacing the heptahelical domain of rat Tas1r3 with that of human TAS1R3 we produced a chimeric receptor that was sensitive for NHDC when coexpressed with rat Tas1r2 (Fig. 1C). In marked contrast, all receptors that contain the entire rat Tas1r3 or its heptahelical domain did not respond to NHDC (Fig. 1C). Notably, although the amplitudes and the response pattern of the receptor chimaeras varied (for details see Ref. 13, supplement) we can demonstrate that all tested receptor chimeras were functional because they could be activated by at least one sweetener (Fig. 1C, D). These results clearly indicate that the heptahelical domain of TAS1R3 appears to be crucially involved in the activation of the sweet receptor by NHDC.

### Receptor activation by NHDC is competitively inhibited by lactisole

**Figure 2:**
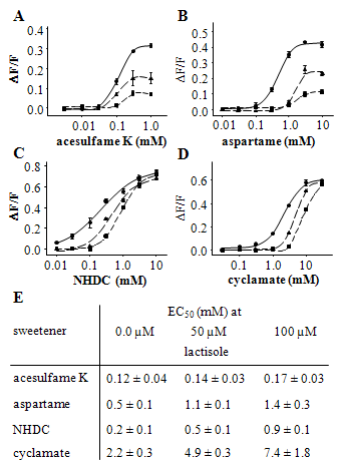
**Inhibition of hTAS1R2/hTAS1R3 by lactisole**. (A-D) Concentration responses of cells transfected with *hTAS1R2*-*hTAS1R3 *DNAs to sweetners mixed with different lactisole concentrations. No lactisole present (filled circles, solid line), 50 μM lactisole (filled triangles up, dashed line), and 100 μM lactisole (filled squares, dash-dotted line). (E) EC_50 _values for acesulfame K, aspartame, NHDC, and cyclamate in the absence or presence of 50 μM and 100 μM lactisole.

Recently, it has been shown that the binding sites for the sweetener cyclamate and the sweet inhibitor lactisole overlap in the heptahelical domain of TAS1R3 [14,15]. We therefore reasoned that the NHDC binding site might also overlap with that for lactisole. In this case one would expect that lactisole competitively inhibits the activation of hTAS1R2/hTAS1R3 by NHDC and cyclamate. In contrast, receptor activation by sweeteners that bind to other sites should be allosterically inhibited. To verify this assumption we recorded concentration-response curves for NHDC, cyclamate, acesulfame K and aspartame in the presence of different concentrations of lactisole in cells expressing hTAS1R2/hTAS1R3 (Fig. 2).

In line with these expectations lactisole did not alter the EC_50 _values for acesulfame K but reduced the signal amplitudes up to ~75% (Fig. 2A, E), suggesting that it is an allosteric inhibitor of acesulfame K. Likewise, lactisole diminished the signal amplitudes induced by aspartame (Fig. 2B), which is consistent with an allosteric inhibition of aspartame-mediated receptor activation. Interestingly, in case of aspartame, the pronounced reductions in signal amplitudes were accompanied by a right-shift in the EC_50 _values (Fig. 2B, E). This may be due to an influence of lactisole on the aspartame binding site by negative cooperativity [20]. In contrast, lactisole did not diminish the signal amplitudes elicited by NHDC or cyclamate (Fig. 2C, D). Instead, lactisole clearly increased the EC_50 _values of these compounds ~fourfold (Fig. 2E). This observed competitive inhibition suggests that the binding sites for lactisole, cyclamate and NHDC overlap in the heptahelical domain of human TAS1R3. Interestingly, we observed slope changes for two of the four tested compounds. While Aspartame and Acesulfam K had constant Hill slope of ~2 the Hill coefficient for NHDC in the presence of 0.0 μM, 50 μM or 100 μM lactisole was 0.8, 1.2 and 1.5, respectively. The calculated Hill coefficient for cyclamate was 1.6, 2.5 and 2.7, respectively. This may be best explained by allosteric effects and may therefore indicate the existence of additional interaction sites either for lactisole or for the sweeteners [21].

### NHDC interacts with residues of the lactisole and cyclamate binding site

Next, we investigated which amino acids of the binding sites for lactisole or cyclamate are critical determinants for receptor activation by NHDC. Therefore, we analyzed the effect of 16 point mutations (Q636A^3.28^, R723A^5.36^, S729A^5.42^, A733V^5.46^, L798I^7.36^, V779A^6.52^, R790Q^ex3^, H734A^5.47^, Q637E^3.29^, H641A^3.33^, S640A^3.32^, H721A^ex2^, F730L^5.43^, W775A^6.48^, F778A^6.51^, L782A^6.55^) in the heptahelical domain of hTAS1R3 that have previously been shown to influence receptor sensitivity to cyclamate or lactisole [14,15] on the sweet receptor's response to NHDC. The effects were measured by recording calcium responses in HEK293T-G16Gust44 cells coexpressing hTAS1R2 and the various hTAS1R3 mutants. Cells expressing the mutant H734A^5.47 ^did not respond to NHDC, cyclamate, aspartame or acesulfame K (Additional file 1). We assumed that this mutation generally impairs the function of the receptor and excluded it from further analyses. Seven receptor mutants (Q636A^3.28^, R723A^5.36^, S729A^5.42^, A733V^5.46^, V779A^6.52^, R790Q^ex3^, L798I^7.36^) showed EC_50 _values for NHDC similar to that of the wild type receptor (Table 1, normal script, Additional file 2), suggesting that these residues have no impact on the action of NHDC on the sweet receptor. The eight remaining TAS1R3 mutants, Q637E^3.29^, S640A^3.32^, H641A^3.33^, H721A^ex2^, F730L^5.43^, W775A^6.48^, F778A^6.51^, and L782A^6.55 ^showed at least eightfold higher EC_50 _values for NHDC than the wild type receptor (Table 1, bold script, Additional file 2). The decreased sensitivities of these eight mutant receptors were specific for NHDC since their EC_50 _values for aspartame were not altered (Table 1). Additionally, immunocytochemical analysis revealed similar expression patterns for all examined TAS1R3 receptor variants (Additional file 3). Thus, the increased EC_50 _values suggest that Q637^3.29^, H641^3.33^, S640^3.32^, H721^ex2^, F730^5.43^, W775^6.48^, F778^6.51^, L782^6.55 ^are important for the activation of the sweet taste receptor by NHDC.

**Table 1:** Effects of various mutations on the activation of the sweet taste receptor by neohesperidin dihydrochalcone (NHDC) and aspartame (ASP)

**variant**	**NHDC EC_50 _(mM)**	**NHDC maximal signal (% of wt)**	**ASP EC_50 _(mM)**	**ASP maximal signal (% of wt)**
wt	0.1 ± 0.06	100	1.3 ± 0.1	100
Q636A^3.28^	0.3 ± 0.2	61 ± 29	1.1 ± 0.1	70 ± 18
**Q637E^3.29^**	**1.5 ± 0.4**	**51 ± 13**	1.7 ± 0.2	51 ± 10
**S640A^3.32^**	**0.8 ± 0.2**	**63 ± 18**	1.4 ± 0.5	61 ± 40
**H641A^3.33^**	**1.2 ± 0.4**	**57 ± 22**	1.8 ± 0.4	79 ± 70
**H721A^ex2^**	**0.8 ± 0.3**	**66 ± 10**	1.6 ± 0.3	60 ± 40
R723A^5.36^	0.2 ± 0.1	69 ± 71	1.5 ± 0.5	68 ± 50
S729A^5.42^	0.2 ± 0.1	80 ± 16	0.9 ± 0.4	72 ± 20
**F730L^5.43^**	**0.9 ± 0.1**	**70 ± 18**	1.7 ± 0.4	87 ± 27
A733V^5.46^	0.1 ± 0.02	133 ± 8	1.1 ± 0.3	92 ± 80
H734A^5.47^	n.f.	--	n.f.	--
**W775A^6.48^**	**>3**	**30 ± 41**	1.9 ± 0.2	20 ± 30
**F778A^6.51^**	**1.5 ± 0.4**	**50 ± 18**	1.2 ± 0.1	80 ± 80
V779A^6.52^	0.2 ± 0.1	88 ± 14	1.2 ± 0.3	52 ± 10
**L782A^6.55^**	**>3**	**50 ± 11**	1.7 ± 0.3	95 ± 90
R790Q^ex3^	0.1 ± 0.06	77 ± 15	0.9 ± 0.4	71 ± 30
L798I^7.36^	0.2 ± 0.2	66 ± 26	0.9 ± 0.4	81 ± 5

Receptor variants that affect the NHDC response are shown in bold. Values are given as average over at least three independent experiments ± SD. n.f., no response to any tested sweetener. (>), EC_50 _not calculable because saturation is not reached (Additional file 2).

### Modeling of the NHDC binding site predicts interactions with additional residues in the heptahelical domain of TAS1R3

**Figure 3:**
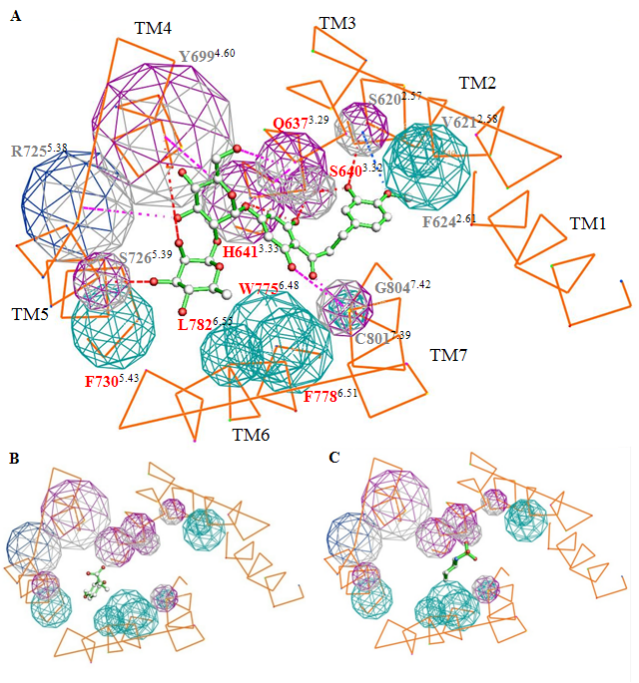
**Three dimensional on top view of the TAS1R3 heptahelical domain binding pocket model**. The binding pocket is docked with NHDC (A), lactisole (B), and cyclamate (C). Intra- and extra cellular loops are removed and the helices are directly connected. The transmembrane segments (TM1–TM7) are denoted in orange. Important residues are labeled in Ballesteros-Weinstein nomenclature in addition to the one letter amino acid code. Amino acids that influence the activation of the sweet receptor by NHDC are shown as spheres. The size of the spheres corresponds to the size of the side chain. The spheres are colored according to their pharmacophoric properties. Hydrophobic amino acids (F, P, M, A, L, I, G, V, W) cyan, H-donor/acceptor (Y, T, S, H, C, N, Q) magenta, H-bond donors with a positive charge (R) blue. Possible H-bond interactions are presented as dotted lines The color of the dotted lines indicate the energy of a H-bond, blue indicates an energy of -0.3–0.6 kcal/Mol, magenta: -0.6–1.2 kcal/Mol, red: -1.2–2.4 kcal/Mol. C atoms of NHDC are displayed in white and oxygen in red. Amino acid positions printed in red refer to residues emerged from the first round of mutational analysis and have been used as anchor points. Amino acid positions printed in grey were predicted from the model to influence receptor activation by NHDC and have been verified by mutational analysis.

To further elucidate the binding mode for NHDC, an alignment of the seven transmembrane helices of the hTAS1R3 with the transmembrane helices of bovine rhodopsin was performed (Additional file 4). Amino acids were extracted at helix positions critical for binding of retinal and then combined in a one dimensional amino acid vector, called ligand pocket vector (LPV) [22]. Amino acids in the TAS1R3 LPV were considered as likely candidates to affect binding of NHDC. Classical homology models imply to have the quality of an X-ray crystal structure and therefore overstress specific, in detail unknown, rotamer conformations of the amino acids. Despite of the substantial progress in building homology models and independently of the level of sophistication of the techniques used, there always remains the risk of a mismatch between optimized rotamers and ligand binding requirements, leading to the rejection of ligands in docking experiments. We therefore designed a simple TM binding pocket pharmacophore (Fig. 3A) to avoid atomic details tending to mask this inherent uncertainty and to concentrate on the general topology of the binding site and the approximate relative orientation of charged, donor/acceptor and neutral side chains. Only amino acid residues are shown which were identified as important for the action of NHDC by site-directed mutagenesis. NHDC was manually docked into the pharmacophore. For the docking process in addition to our results also data from structure-activity relationships of ring substituted dihydrochalcone sweeteners reported by Whitelaw et al. [23,24] were taken into account. These studies reported that the hydroxyl group in the B ring system at C3 of 3'-carboxyhesperitin dihydrochalcone is essential for its sweet taste. Moreover, the introduction of a carboxylic acid at the C3' position in the A ring system enhances the sweetness of the dihydrochalcone structure. Projecting this knowledge onto the chemical structure of NHDC suggests that the OH group in the C3 position is important for receptor-ligand interactions and the C3' position should be in close proximity to H641^3.33 ^(Fig. 3A). Furthermore, H641^3.33 ^was already proposed to interact with the carboxylic acid of lactisole [15] and the mutational studies of the present report indicate that the binding pockets of these two compounds do indeed overlap. The proposed binding mode of NHDC also suggests an interaction of the hydroxyl group at C3 with S640^3.32 ^and the hydroxyl groups at C6' and C2' of NHDC could potentially interact with polar amino acids located in TM3 and TM7, such as H641^3.33^, Q637^3.29^, and C801^7.39 ^respectively (Fig. 3A). In addition, favorable hydrophobic interactions between the B phenyl ring of NHDC could be envisioned with V621^2.58 ^and F624^2.61^, and between the linker of the A and B phenyl ring with L800^7.38 ^and G804^7.42^. Furthermore, the model (Fig. 3A) predicts additional putative interaction sites, such as an interaction between the C3-hydroxylgroup of NHDC with S620^2.57 ^in TM2. Moreover, the polar sugar moieties of NHDC could be interacting with polar amino acids of TM4 and TM5, e.g. Y699^4.60^, T724^5.37^, R725^5.38 ^and S726^5.39^.

### Validation of the model by functional analysis of additional mutants

To verify the predictions of the model we selected 10 additional amino acids in hTAS1R3 (S620^2.57^, V621^2.58^, F624^2.61^, Y699^4.60^, T724^5.37^, R725^5.38^, S726^5.39^, L800^7.38^, C801^7.39^, and G804^7.42^) as candidates that might also influence the activation of the sweet receptor by NHDC. To test their impact on the receptor function we created fourteen hTAS1R3 variants (S620A^2.57^, V621L^2.58^, V621I^2.58^, F624L^2.61^, Y699L^4.60^, Y699F^4.60^, T724L^5.37^, R725M^5.38^, S726A^5.39^, L800F^7.38^, C801I^7.39^, C801L^7.39^, G804A^7.42^, and G804V^7.42^) and tested their responses to NHDC by calcium imaging after coexpression with hTAS1R2 in HEK293T-G16Gust44 cells. The results show that the mutant receptors V621L^2.58 ^and C801L^7.39 ^could not be activated by NHDC, cyclamate, aspartame and acesulfame K (Table 2, Additional file 1), suggesting that these mutants are not functional. Therefore, we excluded them from further analyses. The receptor mutant L800F^7.38 ^responded to NHDC in a manner similar to the wild type receptor (Table 2, Additional file 2), suggesting that L800 is not important for receptor activation by NHDC. Ten receptor mutants (S620A^2.57^, V621I^2.58^, F624L^2.61^, Y699L^4.60^, Y699F^4.60^, T724L^5.37^, R725M^5.38^, S726A^5.39^, C801I^7.39^, and G804A^7.42^) had five to sixteen fold increased EC_50 _values for NHDC (Table 2, bold script, Additional file 2) and one mutant, G804V^7.42^, did not respond to NHDC at any tested concentration (Table 2, Additional file 1 and 2). The effects of these mutants were specific for NHDC because they all showed similar EC_50 _values for aspartame (Table 2). Moreover, the functional differences were not correlated with different expression levels or reduced signal amplitudes (Table 2, Additional file 3). The changes in the EC_50 _values suggest that these mutations directly affect the activation of the sweet taste receptor by NHDC, thus validating our model.

**Table 2:** Effect of mutations predicted by molecular modeling on the activation of the sweet taste receptor by neohesperidin dihydrochalcone (NHDC) and aspartame (ASP)

**variant**	**NHDC EC_50 _(mM)**	**NHDC maximal signal (% of wt)**	**ASP EC_50 _(mM)**	**ASP maximal signal (% of wt)**
wt	0.1 ± 0.06	100	1.3 ± 0.1	100
**S620A^2.57^**	**1.3 ± 0.5**	**55 ± 80**	1.2 ± 0.3	64 ± 4
V621L^2.58^	n.f.	---	n.f.	---
**V621I^2.58^**	**>1**	**60 ± 14**	1.9 ± 0.4	61 ± 1
**F624L^2.61^**	**>1**	**35 ± 70**	1.4 ± 0.2	21 ± 4
**Y699L^4.60^**	**0.7 ± 0.1**	**83 ± 40**	1.6 ± 0.4	78 ± 9
**Y699F^4.60^**	**0.6 ± 0.4**	**72 ± 17**	1.2 ± 0.3	56 ± 2
**T724L^5.37^**	**0.5 ± 0.3**	**58 ± 60**	1.5 ± 0.2	69 ± 2
**R725M^5.38^**	**1.3 ± 0.4**	**64 ± 14**	1.5 ± 0.7	56 ± 1
**S726A^5.39^**	**0.9 ± 0.3**	**60 ± 50**	0.9 ± 0.3	50 ± 2
L800F^7.38^	0.2 ± 0.1	91 ± 70	1.2 ± 0.3	90 ± 1
**C801I^7.39^**	**1.6 ± 0.7**	**70 ± 13**	1.2 ± 0.5	50 ± 9
C801L^7.39^	n.f.	---	n.f.	---
**G804A^7.42^**	**1.3 ± 0.5**	**53 ± 10**	1.6 ± 0.2	44 ± 1
**G804V^7.42^**	**n.r**.	**---**	1.3 ± 0.3	21 ± 2

Receptor variants that affect the NHDC response are shown in bold. Values are given as average over at least three independent experiments ± SD. n.f. no response to any tested sweetener, n.r. no response to neohesperidin dihydrochalcone up to highest tested concentration (6 mM), but responses to other sweeteners. (>), EC_50 _not calculable because saturation is not reached (Additional file 2).

### Some of the newly identified residues also influence the interaction of the sweet receptor with lactisole or cyclamate

**Figure 4:**
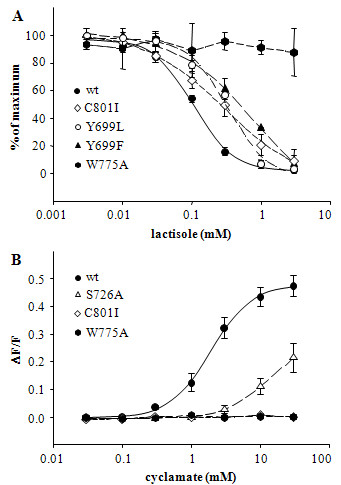
**Identification of additional determinants in TAS1R3 critical for responsiveness to cyclamate and lactisole**. (A) Concentration-dependent inhibition of calcium responses to 10 mM aspartame by lactisole in HEK293T-G16Gust44 cells cotransfected with DNA for wild type hTAS1R2/hTAS1R3 (filled circle, solid line), C801I^7.39 ^(open diamond, short dashed line), Y699L^5.60 ^(open circle, dash-dot-dotted line), Y699F^5.60 ^(filled triangle up, medium dashed line), or W775A^6.48 ^(filled hexagon, dash-dotted line) and hTAS1R2 DNA. (B) Concentration-dependent responses of HEK293T-G16Gust44 cells to cyclamate cotransfected with DNA for hTAS1R3 (filled circle, solid line), S726A^5.39 ^(open triangle up, medium dashed line), C801I^7.39 ^(open diamond, short dashed line), or W775A^6.48 ^(filled hexagon, dash-dotted line) and hTAS1R2.

Since our results indicate a partial overlap of the NHDC binding site with those for lactisole and cyclamate, we next asked, if any of the newly identified residues also affect the sweet receptor's responses to these compounds. To this end, we tested all mutant hTAS1R3 subunits by coexpression with hTAS1R2 in HEK293T-G16Gust44 cells for their sensitivity to lactisole and cyclamate (Additional file 1). While we essentially confirmed the findings of Jiang et al. [14,15] that amino acids in positions 636^3.28^, 637^3.29^, 640^3.32^, 641^3.33^, 721^ex2^, 723^5.36^, 729^5.42^, 730^5.43^, 733^5.46^, 778^6.51^, 779^6.52^, 782^6.55^, 790^ex3^, and 798^7.36 ^of hTAS1R3 contribute to the sensitivity of the sweet receptor to cyclamate and/or lactisole, we found additional amino acids in the heptahelical domain of hTAS1R3 that altered the responses of the sweet receptor to the two compounds. The mutant receptor W775A^6.48 ^failed to respond to lactisole (Fig. 4A), suggesting that this residue is crucial for the sensitivity of the sweet receptor to the inhibitor. The mutants C801I^7.39 ^(IC_50 _= 0.3 ± 0.01 mM), Y699L^4.60 ^(IC_50 _= 0.3 ± 0.01 mM), and Y699F^4.60 ^(IC_50 _= 0.5 ± 0.01 mM) showed three- to fivefold reduced responses to lactisole compared to the wild type receptor (IC_50 _= 0.1 ± 0.01 mM). Thus, we conclude that the amino acid positions Y699^4.60 ^and C801^7.39 ^also contribute to the inhibition of the sweet receptor by lactisole. The mutants C801I^7.39 ^and W775A^6.48 ^could not be activated at any tested concentration of cyclamate indicating the importance of these residues for the sweet receptor's responsiveness to cyclamate (Fig. 4B, Additional file 1). In addition, the mutant receptor S726A^5.39 ^displayed more than fivefold lower sensitivity to cyclamate (EC_50 _> 10 mM) than the wild type receptor (EC_50 _= 1.9 ± 0.1 mM), suggesting that all three residues contribute to the receptor's ability to be activated by cyclamate.

## Discussion

### NHDC binds in the heptahelical domain of TAS1R3

Several independent lines of evidence clearly demonstrate that NHDC interacts with specific amino acid residues in the heptahelical domain of the TAS1R3 subunit. First, lactisole, which interacts with the heptahelical domain of TAS1R3 [13,15], inhibited the activation of hTAS1R2/hTAS1R3 by NHDC competitively, while sweeteners that interact with other parts of the receptor were allosterically inhibited (Fig. 2). Second, the functional analysis of rat/human TAS1R2/TAS1R3 chimeric receptors showed that NHDC activated all chimeric receptors that contain the heptahelical domain of human TAS1R3. In contrast, all chimeras that contained the heptahelical domain of rat Tas1r3 could not be activated by NHDC (Fig. 1). Third, seventeen amino acid exchanges in the heptahelical domain of hTAS1R3 specifically reduced the NHDC responses but did not influence the responses to aspartame (Table 1 + 2), which interacts with the extracellular N-terminal domain of TAS1R2 [13]. Moreover, the finding that NHDC activates the human sweet receptor but not the rat sweet taste taste receptor delivers a molecular basis for the well-known lack of NHDC preference in rodents [25,26]. In addition, our results confirm previous findings that functional differences between rat and human sweet receptor [4] are caused by sequence variations in the heptahelical domain of TAS1R3 [13,14,16].

### Elucidation of the NHDC binding site

To further understand the interaction between NHDC and the heptahelical domain of hTAS1R3 we established a simple 3D receptor pharmacophore model of the NHDC binding site. Notably, in the model each amino acid residue in the heptahelical domain of the TAS1R3 receptor is identified by its position. Moreover, the numbering system proposed by Ballesteros and Weinstein [27] is shown as superscripts to facilitate the comparison with results of other GPCRs. The model considers results obtained from our functional studies, available data about the suggested binding modes of cyclamate and lactisole [14,15], information about conserved amino acids in GPCRs [27], and sensory studies of NHDC derivatives [23,24]. These sensory studies have shown that the hydroxyl group in C3 of the B ring is crucial for the sweet taste of dihydrochalcones [23,28]. In line with these findings, our receptor model proposes that this hydroxyl group can form hydrogen bonds with either of the two serine residues in positions 640^3.32 ^of TM3 and 620^2.57 ^of TM2. The possible interaction of the C3 hydroxyl group with both serines can explain why mutations at either position strongly reduced but not completely abolished the responses of the receptor to NHDC.

The model also predicts that H641^3.33^, Q637^3.29^, and C801^7.39 ^can form hydrogen bonds with hydroxyl groups in the A ring as suggested by the decreased EC_50 _values for NHDC of the corresponding mutants. In addition, our model predicts that hydroxyl groups of the sugar moieties interact with Y699^4.60^, R725^5.38^, and S726^5.39 ^in TM4 and TM5. Indeed, mutations in these positions impaired the interaction of NHDC with the receptor. Notably, the aglycon hesperitin dihydrochalcone that lacks the sugar rings also tastes sweet [29,30]. Therefore, it might be conceivable that amino acids at these three positions are less important for the activation of the sweet taste receptor by NHDC, but rather enhance its affinity. Furthermore, the methoxy group at position C4 of the B ring is in close proximity to V621^2.58 ^and F624^2.61 ^and is therefore likely engaged in favorable hydrophobic interactions with the side chains of these amino acids as concluded from the shift in EC_50 _values for NHDC at the corresponding mutant receptors.

### The binding sites for NHDC, cyclamate and lactisole share amino acid residues

**Figure 5:**
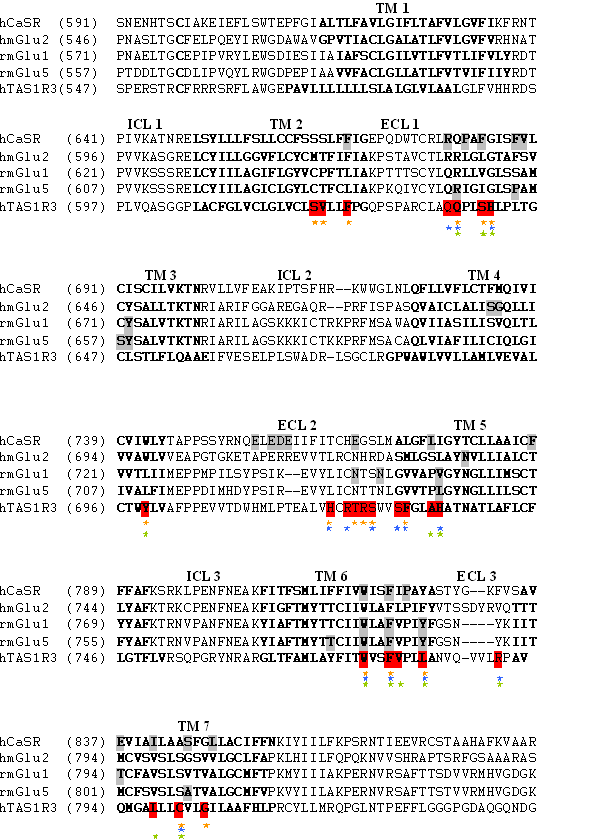
**Alignment of the heptahelical domain of hCaSR, hmGlu2, rmGlu1, rmGlu5, and hTAS1R3**. Transmembrane segments (TM) are shown in bold. Intra- and extracellular loops are marked by ICL and ECL, respectively. Amino acids that influence allosteric modulator activity in hCaSR, hmGlu2, rmGlu1 and 5 are marked in grey. Positions that alter the response of the sweet receptor to lactisole, cyclamate, or NHDC are shown in red. Asterisks indicate residues involved in receptor activation by NHDC (orange) or cyclamate (blue). Green asteriks denote residues mediating sensitivity of the receptor to lactisole.

The sweet inhibitor lactisole, which interacts with specific residues in the heptahelical domain of TAS1R3 [13,15,16], inhibited the activation of the sweet receptor by NHDC and cyclamate competitively (Fig. 2). These results suggest that the binding sites of lactisole, cyclamate, and NHDC overlap. Interestingly, the observed competitive effects between NHDC and lactisole (Fig. 2) may also explain why lactisole in contrast to many other sweeteners did not inhibit the sweet taste of NHDC in humans [31]. A previous report of Jiang et al. proposed that cyclamate and lactisole interact with a common set of amino acid residues[14,15]. Our findings confirm this observation by showing that seven mutations (Q637^3.29^, H641^3.33^, W775^6.48^, F778^6.51^, L782^6.55^, R790Q^ex3^, and C801^7.39^) influence both, the lactisole mediated inhibition and the cyclamate induced activation of the sweet receptor (Fig. 5, Table 1 + 2). While cyclamate and lactisole only use parts of the TAS1R3 pharmacophore, our model predicts that NHDC uses most of it due to its larger size (Fig. 3). In line with these predictions, eight of the seventeen amino acids that alter receptor activation by NHDC (Q637^3.29^, S640^3.32^, H641^3.33^, Y699^4.60^, W775^6.48^, F778^6.51^, L782^6.55^, and C801^7.39^), also influence lactisole-mediated inhibition of the receptor. Similarly, nine of the seventeen residues (Q637^3.29^, H641^3.33^, H721^ex2^, S726^5.39^, F730^5.43^, W775^6.48^, F778^6.51^, L782^6.55^, and C801^7.39^) mediate activation by cyclamate, while six (Q637^3.29^, H641^3.33^, W775^6.48^, F778^6.51^, L782^6.55^, and C801^7.39^) influence receptor inhibition by lactisole as well as receptor activation by cyclamate (Fig. 5, Table 1 + 2).

Our mutational analysis identified four additional amino acid residues that have not previously been recognized (Y699^4.60^, S726^5.39^, W775^6.48^, and C801^7.39^) as interaction partners for lactisole and cyclamate [14,15]. These residues nicely refine the model proposed by Jiang et al. by enabling additional interactions between the two compounds and TAS1R3. This finding supports the validity of our model and suggests that it is compatible with that of Jiang et al.

Transmembrane domains six and seven are generally involved in GPCR activation [32-34]. The following observations suggest that the four residues W775A^6.48^, F778A^6.51^, L782A^6.55^, and C801I^7.39 ^of TM6 and TM7 could participate in conformational changes of the sweet taste receptor from the ground to an active state and vice versa. Firstly, F778^6.51 ^and W775^6.48 ^are highly conserved across class A and class C GPCRs [14,15,35]. Secondly, our study showed that mutations at these positions alter the receptor's sensitivities to the agonists NHDC and cyclamate as well as to the inhibitor lactisole. Thirdly, Jiang et al. also found that mutations in position L782A^6.55 ^abolished activation of the sweet taste receptor by cyclamate and enhanced the receptor's responsiveness to the inhibitor lactisole [15].

The amino acid residue C801^7.39 ^in TM7 also affects the sensitivity of the sweet taste receptor to cyclamate, NHDC and lactisole. While it is in the proximity of the NHDC and cyclamate binding sites, it is quite remote from that of lactisole (Fig. 3A, B, C). This argues against a direct interaction of lactisole with C801^7.39^. It is therefore conceivable that in addition to W775^6.48^, F778^6.51^, and L782^6.55 ^in TM6, C801^7.39 ^in TM7 may also play a general role in the activation process of the sweet taste receptor. This assumption is further supported by the observation that amino acids in TM7 of the calcium sensing receptor such as V836^7.31^, Q837^7.32 ^and A843^7.38 ^play a key role in the activation of this receptor [32].

Notably, aspartame, which likely interacts with the N-terminal domain of hTAS1R2 [12,13] could activate the mutants W775^6.48^, F778^6.51^, L782^6.55 ^and C801I^7.39 ^in a manner, similar to the wild type receptor (Tab. 1 and 2). Therefore, it might be possible that tastants that bind to different receptor sites elicit different types of active confirmations as has been observed before for the activation of the β_2_-adrenergic receptor by salbutamol and catechol [36].

### The NHDC binding site overlaps with the binding sites of allosteric modulators in other class C GPCRs

Sequence comparisons revealed that nine of the amino acid positions in hTAS1R3 that influence the sensitivity of the sweet receptor to NHDC, cyclamate or lactisole correspond to residues that are responsible for binding allosteric modulators in the calcium sensing receptor and metabotropic glutamate receptors (Fig. 5) [32-34,37-43]. This suggests a general role for these positions in allosterism in this class of receptors and shows that positions of allosteric binding sites are partly conserved amongst class C GPCRs. Thus, critical determinants found in non-taste receptors are good candidates to uncover additional binding sites for sweet and umami tasting substances or modulators.

## Conclusion

Our mutational analysis combined with functional studies and molecular modeling identified the binding pocket of NHDC in the heptahelical domain of human TAS1R3. We found that the binding pocket of NHDC overlaps with those of the sweetener cyclamate and the sweet taste inhibitor lactisole. Seven of the amino acid positions crucial for activation of hTAS1R2/hTAS1R3 by neohesperidin dihydrochalcone are involved in the binding of allosteric modulators of other class C GPCRs. This suggests a general role of these amino acid positions in allosterism and points to a common architecture of the heptahelical domains of class C GPCRs.

## Methods

### Ballesteros and Weinstein nomenclature

The position of each amino acid residue in the heptahelical domain of TAS1R3 is identified both by its position and by the generic numbering system proposed by Ballesteros and Weinstein [27] shown as superscripts. In the Ballesteros-Weinstein nomenclature the most conserved residue in each helix is given the number 50. This is N1.50, D2.50, R3.50, W4.50, P5.50, P6.50 and P7.50 in transmembrane helix 1–7 of the rhodopsin receptor. The corresponding amino acids in the TAS1R3 are given the same numbers (Additional file 4).

### Construction of TAS1R3 mutants

Human TAS1R3 receptor mutants were generated by site directed mutagenesis according to the QuickChange protocol (Stratagene, La Jolla, CA). The forward and reverse primers contained the desired mutations and annealed to the same sequence on opposite strands of the plasmids. The following TAS1R3 receptor mutants were generated: S620A^2.57^, V621L^2.58^, V621I^2.58^, F624L^2.61^, Q636A^3.28^, Q637E^3.29^, S640A^3.32^, H641A^3.33^, Y699L^4.60^, Y699F^4.60^, H721A^ex2^, R723A^5.36^, T724L^5.37^, R725M^5.38^, S726A^5.39^, S729A^5.42^, F730L^5.43^, A733V^5.46^, H734A^5.47^, W775A^6.48^, F778A^6.51^, V779A^6.52^, L782A^6.55^, R790Q^ex3^, L798I^7.36^, L800F^7.38^, C801L^7.39^, C801I^7.39^, G804V^7.42^, and G804A^7.42^.

The position of each amino acid residue in the seven heptahelical domain of the TAS1R3 receptor is identified both by its position and by the generic numbering system proposed by Ballesteros and Weinstein [27] shown as superscripts.

### Functional expression

The cDNAs for TAS1Rs tagged at the C-terminus with the herpes simplex virus glycoprotein D (HSV) were transiently transfected into HEK293T cells stably expressing the chimeric G-protein subunit G16Gust44 [44] using Lipofectamine 2000 (Invitrogen, Carlsbad, CA) according to the manufacturer's protocol. 3–4 hours after transfection, DMEM was replaced by low-glucose DMEM supplemented with GlutaMAX and 10% dialyzed FBS (Invitrogen, Carlsbad, CA). 24 h post transfection, cells were loaded for 1 h with the calcium sensitive dye Fluo4-AM (2 μg/ml in DMEM, Molecular Probes, Carlsbad, CA). Cells were washed 3x in solution C1 (130 mM NaCl, 5 mM KCl, 10 mM Hepes, 2 mM CaCl_2, _and 5 mM Glucose, pH 7,4). We monitored calcium mobilization following receptor stimulation with sweet tastants by an automated fluorometric imaging plate reader (FLIPR, Molecular Devices, Sunnyvale, CA). Compounds used as stimuli (Sigma-Aldrich, St. Louis, MO; Merck, Whitehouse Station, NJ) were dissolved in C1 solution. All data were collected from at least three independent experiments carried out in duplicate. The obtained calcium signals were corrected for the response of mock transfected cells and normalized to the fluorescence of cells prior to the application of the stimulus using ΔF/F = (F-F0)/F0. Concentration-response curves and EC_50 _and IC_50 _values were calculated in SigmaPlot by nonlinear regression using the function f = ((a-d)/(1+(x/EC50)^nH^)+d) and f = (a-b)/[1+(x/IC50)^*n*H^]+b, respectively.

### Immuncytochemistry

HEK293T-G16Gust44 cells were seeded on poly-D-lysine coated coverslips (10 μg/ml) and transfected with the respective cDNAs. 24 h post transfection cells were washed with PBS and fixed and permeabilized for 5 min in acetone/methanol (1:1). Incubating the cells in 5% goat serum for 30 min reduced non-specific binding. To detect the receptors, antiserum against the HSV-epitope (mouse anti-HSV (Novagen, Madison, WI), 1:10000 in 3% goat serum) was added to the cells for 1 h at room temperature (RT). After washing the cells three times with PBS we added Alexa488-conjugated goat antiserum against mouse IgG (Molecular Probes, Carlsbad, CA), 1:1000 in 3% goat serum) for 1 h at RT. The cells were embedded in Fluorescent Mounting Medium (Dako, Glostrup, Denmark) and analyzed using a fluorescence microscope (Zeiss Axioplan, Jena, Germany) and a camera (RT Slide, Visitron Systems, Munich, Germany).

### TAS1R3 modeling

All modeling calculations were made on a Silicon Graphics Octane with a single R12000 processor using our in-house modeling package Moloc [45]. The alignment of the seven transmembrane helices of TAS1R3 receptor (SWISS-PROT: TS1R3_HUMAN, Q7RTX0) with the transmembrane helices of bovine rhodopsin (pdb ref code 1f88) and the 3D receptor-based pharmacophore modeling was described previously [22]. Briefly, the method relies on a robust alignment algorithm based on conservation indices, focusing on pharmacophore-like relationships between amino acids. Analysis of conservation patterns across the GPCR family and alignment to the rhodopsin x-ray structure (pdb ref code 1f88, Additional file 4) allows the extraction of the amino acids lining the TM binding pocket in a so-called ligand binding pocket vector. In a second step, LPVs are translated to simple 3D receptor pharmacophore models, where a single spherical pharmacophore feature represents each amino acid and all atomic detail is omitted. The pharmacophores are colored according to the pharmacophoric properties of the amino acids. Hydrophobic amino acids (aromatic/aliphatic; F, P, M, A, L, I, G, V, W) are represented by cyan pharmacophores, amino acids with H-donor/acceptor functionalities (Y, T, S, H, C, N, Q) by magenta pharmacophores, and amino acids with H-bond donor functionalities and positive charge (K, R) by blue ones. The pharmacophores with H-bond donor or acceptor properties have in addition a cone, which gives the most likely direction of the H-bond. The new reported receptor-based pharmacophore modeling methodology allows a visual analysis of the general ligand binding properties of any GPCR without the need to concentrate on atomic details of side chain orientations [22]. NHDC is manually docked into the pharmacophores in the TM pocket. Only pharmacophores are shown which were identified as important for the action of NHDC by site-directed mutagenesis.

## Abbreviations

NHDC – neohesperidin dihydrochalcone

GPCR – G-protein coupled receptor

TAS1R – taste receptor family one

HEK293T-G16Gust44 – human embryonal kidney cell line stably expressing the large T antigen and the G-protein chimera G16Gust44

EC_50 _– half maximal effective concentration

IC_50 _– half maximal inhibitory concentration

FLIPR – fluorometric imaging plate reader

## Competing interests

The author(s) declares that there are no competing interests.

## Authors' contributions

MW cloned most of the TAS1R subunits, constructed the chimeric TAS1R3 receptors, carried out the functional and histological studies and participated in the study design. BB coordinated the study design, hold and organized the external collaborations and drafted the manuscript. NAK did the molecular modeling and suggested several sites for receptor mutation. JPS participated in the study design. WM conceived the study, participated in its design, and helped to draft the manuscript. All authors read and approved the final manuscript.

## Supplementary Material

Additional file 1 Activity of all mutants towards different sweeteners. Click here for file

Additional file 2 Concentration-response curves of all mutants towards NHDC. Click here for file

Additional file 3 Expression of hTAS1R3 variants. Click here for file

Additional file 4 Sequence alignment of human TAS1R3 and bovine rhodopsin transmembrane segments (TM). Click here for file

## References

[B1] Montmayeur JP, Liberles SD, Matsunami H, Buck LB (2001). A candidate taste receptor gene near a sweet taste locus. Nat Neurosci.

[B2] Damak S, Rong M, Yasumatsu K, Kokrashvili Z, Varadarajan V, Zou S, Jiang P, Ninomiya Y, Margolskee RF (2003). Detection of sweet and umami taste in the absence of taste receptor T1r3. Science.

[B3] Hoon MA, Adler E, Lindemeier J, Battey JF, Ryba NJ, Zuker CS (1999). Putative mammalian taste receptors: a class of taste-specific GPCRs with distinct topographic selectivity. Cell.

[B4] Li X, Staszewski L, Xu H, Durick K, Zoller M, Adler E (2002). Human receptors for sweet and umami taste. Proc Natl Acad Sci U S A.

[B5] Nelson G, Hoon MA, Chandrashekar J, Zhang Y, Ryba NJ, Zuker CS (2001). Mammalian sweet taste receptors. Cell.

[B6] Max M, Shanker YG, Huang L, Rong M, Liu Z, Campagne F, Weinstein H, Damak S, Margolskee RF (2001). Tas1r3, encoding a new candidate taste receptor, is allelic to the sweet responsiveness locus Sac. Nat Genet.

[B7] Terrillon S, Bouvier M (2004). Roles of G-protein-coupled receptor dimerization. EMBO Rep.

[B8] Nelson G, Chandrashekar J, Hoon MA, Feng L, Zhao G, Ryba NJ, Zuker CS (2002). An amino-acid taste receptor. Nature.

[B9] Brouwer JN, Hellekant G, Kasahara Y, van der Wel H, Zotterman Y (1973). Electrophysiological study of the gustatory effects of the sweet proteins monellin and thaumatin in monkey, guinea pig and rat. Acta Physiol Scand.

[B10] Sclafani A, Abrams M (1986). Rats show only a weak preference for the artificial sweetener aspartame. Physiol Behav.

[B11] Sclafani A, Perez C (1997). Cypha [propionic acid, 2-(4-methoxyphenol) salt] inhibits sweet taste in humans, but not in rats. Physiol Behav.

[B12] Jiang P, Ji Q, Liu Z, Snyder LA, Benard LM, Margolskee RF, Max M (2004). The cysteine-rich region of T1R3 determines responses to intensely sweet proteins. J Biol Chem.

[B13] Xu H, Staszewski L, Tang H, Adler E, Zoller M, Li X (2004). Different functional roles of T1R subunits in the heteromeric taste receptors. Proc Natl Acad Sci U S A.

[B14] Jiang P, Cui M, Zhao B, Snyder LA, Benard LM, Osman R, Max M, Margolskee RF (2005). Identification of the cyclamate interaction site within the transmembrane domain of the human sweet taste receptor subunit T1R3. J Biol Chem.

[B15] Jiang P, Cui M, Zhao B, Liu Z, Snyder LA, Benard LM, Osman R, Margolskee RF, Max M (2005). Lactisole interacts with the transmembrane domains of human T1R3 to inhibit sweet taste. J Biol Chem.

[B16] Winnig M, Bufe B, Meyerhof W (2005). Valine 738 and lysine 735 in the fifth transmembrane domain of rTas1r3 mediate insensitivity towards lactisole of the rat sweet taste receptor. BMC Neurosci.

[B17] Dogan M (2002). Neohesperidin DC in food products: ; Trabzon, Türkiye..

[B18] DuBois GE, Crosby GA, Stephenson RA, Wingard RE (1977). Dihydrochalcone sweeteners. Synthesis and sensory evaluation of sulfonate derivatives. J Agric Food Chem.

[B19] DuBois GE, Crosby GA, Stephenson RA (1981). Dihydrochalcone sweeteners. A study of the atypical temporal phenomena. J Med Chem.

[B20] Durroux T (2005). Principles: a model for the allosteric interactions between ligand binding sites within a dimeric GPCR. Trends Pharmacol Sci.

[B21] Morini G, Bassoli A, Temussi PA (2005). From small sweeteners to sweet proteins: anatomy of the binding sites of the human T1R2_T1R3 receptor. J Med Chem.

[B22] Kratochwil NA, Malherbe P, Lindemann L, Ebeling M, Hoener MC, Muhlemann A, Porter RH, Stahl M, Gerber PR (2005). An automated system for the analysis of G protein-coupled receptor transmembrane binding pockets: alignment, receptor-based pharmacophores, and their application. J Chem Inf Model.

[B23] Whitelaw ML, Chung HJ, Daniel JR (1991). Synthesis and sensory evaluation of ring-substituted dihydrochalcone sweeteners. 2. Analogues of 3'-Carboxyhesperetin dihydrocahlcone, a high-potency dihydrochalcone sweetener. J Agric Food Chem.

[B24] Whitelaw ML, Daniel JR (1991). Synthesis and sensory evaluation of ring-substituted dihydrochalcone sweeteners. J Agric Food Chem.

[B25] Naim M, Rogatka H, Yamamoto T, Zehavi U (1982). Taste responses to neohesperidin dihydrochalcone in rats and baboon monkeys. Physiol Behav.

[B26] Bachmanov AA, Tordoff MG, Beauchamp GK (2001). Sweetener preference of C57BL/6ByJ and 129P3/J mice. Chem Senses.

[B27] Ballesteros JA, H. W (1995). Integrated Methods for the Construction of Three-Dimensional Models and Computational Probing of Structure-Function Relations in G-Protein-Coupled Receptors.. Methods in Neuroscience.

[B28] Bassoli A, Merlini L, Morini G (2002). Isovanillyl sweeteners. From molecules to receptors. Pure Appl Chem.

[B29] DuBois GE, Crosby GA, Saffron P (1977). Nonnutritive sweeteners: taste-structure relationships for some new simple dihydrochalcones. Science.

[B30] Horowitz RM, Gentili B (1960). Flavonoids of the Ponderosa lemon. Nature.

[B31] Schiffman SS, Booth BJ, Sattely-Miller EA, Graham BG, Gibes KM (1999). Selective inhibition of sweetness by the sodium salt of +/-2-(4-methoxyphenoxy)propanoic acid. Chem Senses.

[B32] Hu J, McLarnon SJ, Mora S, Jiang J, Thomas C, Jacobson KA, Spiegel AM (2005). A region in the seven-transmembrane domain of the human Ca2+ receptor critical for response to Ca2+. J Biol Chem.

[B33] Malherbe P, Kratochwil N, Knoflach F, Zenner MT, Kew JN, Kratzeisen C, Maerki HP, Adam G, Mutel V (2003). Mutational analysis and molecular modeling of the allosteric binding site of a novel, selective, noncompetitive antagonist of the metabotropic glutamate 1 receptor. J Biol Chem.

[B34] Petrel C, Kessler A, Maslah F, Dauban P, Dodd RH, Rognan D, Ruat M (2003). Modeling and mutagenesis of the binding site of Calhex 231, a novel negative allosteric modulator of the extracellular Ca(2+)-sensing receptor. J Biol Chem.

[B35] Pin JP, Galvez T, Prezeau L (2003). Evolution, structure, and activation mechanism of family 3/C G-protein-coupled receptors. Pharmacol Ther.

[B36] Swaminath G, Deupi X, Lee TW, Zhu W, Thian FS, Kobilka TS, Kobilka B (2005). Probing the beta2 adrenoceptor binding site with catechol reveals differences in binding and activation by agonists and partial agonists. J Biol Chem.

[B37] Hu J, Reyes-Cruz G, Chen W, Jacobson KA, Spiegel AM (2002). Identification of acidic residues in the extracellular loops of the seven-transmembrane domain of the human Ca2+ receptor critical for response to Ca2+ and a positive allosteric modulator. J Biol Chem.

[B38] Malherbe P, Kratochwil N, Zenner MT, Piussi J, Diener C, Kratzeisen C, Fischer C, Porter RH (2003). Mutational analysis and molecular modeling of the binding pocket of the metabotropic glutamate 5 receptor negative modulator 2-methyl-6-(phenylethynyl)-pyridine. Mol Pharmacol.

[B39] Miedlich SU, Gama L, Seuwen K, Wolf RM, Breitwieser GE (2004). Homology modeling of the transmembrane domain of the human calcium sensing receptor and localization of an allosteric binding site. J Biol Chem.

[B40] Pagano A, Ruegg D, Litschig S, Stoehr N, Stierlin C, Heinrich M, Floersheim P, Prezeau L, Carroll F, Pin JP, Cambria A, Vranesic I, Flor PJ, Gasparini F, Kuhn R (2000). The non-competitive antagonists 2-methyl-6-(phenylethynyl)pyridine and 7-hydroxyiminocyclopropan[b]chromen-1a-carboxylic acid ethyl ester interact with overlapping binding pockets in the transmembrane region of group I metabotropic glutamate receptors. J Biol Chem.

[B41] Petrel C, Kessler A, Dauban P, Dodd RH, Rognan D, Ruat M (2004). Positive and negative allosteric modulators of the Ca2+-sensing receptor interact within overlapping but not identical binding sites in the transmembrane domain. J Biol Chem.

[B42] Ray K, Tisdale J, Dodd RH, Dauban P, Ruat M, Northup JK (2005). Calindol, a positive allosteric modulator of the human Ca(2+) receptor, activates an extracellular ligand-binding domain-deleted rhodopsin-like seven-transmembrane structure in the absence of Ca(2+). J Biol Chem.

[B43] Schaffhauser H, Rowe BA, Morales S, Chavez-Noriega LE, Yin R, Jachec C, Rao SP, Bain G, Pinkerton AB, Vernier JM, Bristow LJ, Varney MA, Daggett LP (2003). Pharmacological characterization and identification of amino acids involved in the positive modulation of metabotropic glutamate receptor subtype 2. Mol Pharmacol.

[B44] Ueda T, Ugawa S, Yamamura H, Imaizumi Y, Shimada S (2003). Functional interaction between T2R taste receptors and G-protein alpha subunits expressed in taste receptor cells. J Neurosci.

[B45] Molecular design software. http://www.moloc.ch.

