# Supplementary figures and images for: The binding site for neohesperidin dihydrochalcone at the human sweet taste receptor
Source: BMC Struct Biol. 2007 Oct 12;7:66. doi: 10.1186/1472-6807-7-66 (PMC2099433; doi:10.1186/1472-6807-7-66)

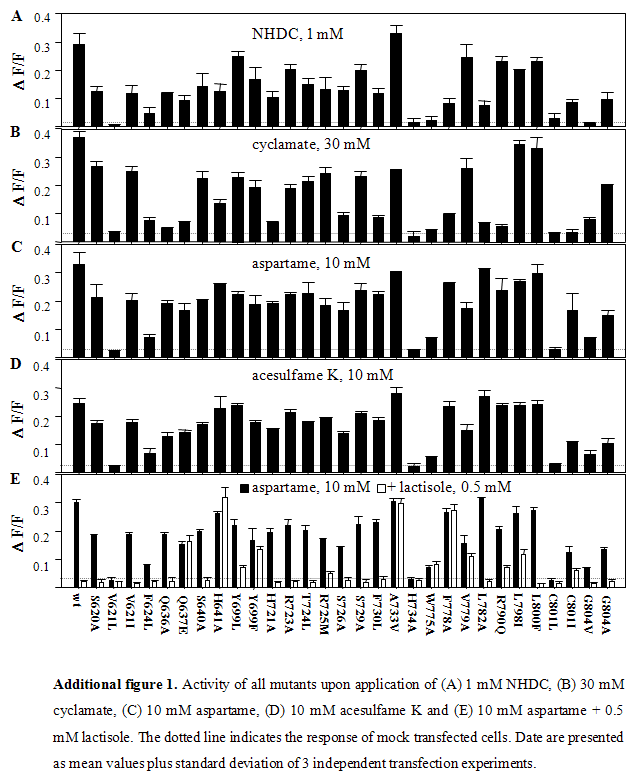

Supplement: Additional file 1 — Activity of all mutants towards different sweeteners. [file 1472-6807-7-66-S1.tiff]

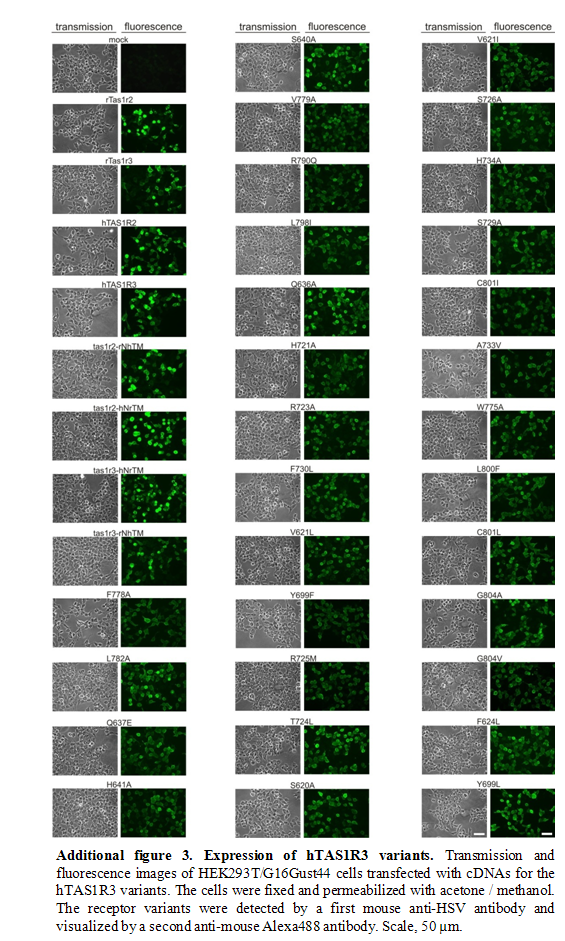

Supplement: Additional file 3 — Expression of hTAS1R3 variants. [file 1472-6807-7-66-S3.tiff]

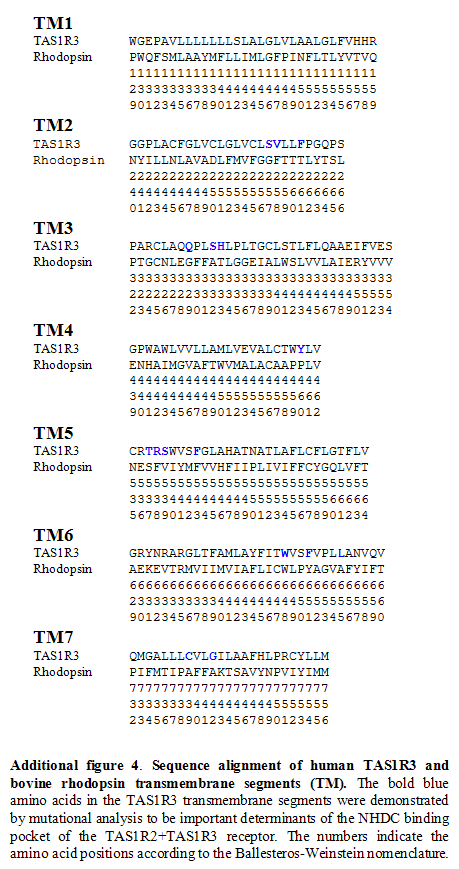

Supplement: Additional file 4 — Sequence alignment of human TAS1R3 and bovine rhodopsin transmembrane segments (TM). [file 1472-6807-7-66-S4.tiff]
