# Supplementary material for: The binding site for neohesperidin dihydrochalcone at the human sweet taste receptor
Source: BMC Struct Biol. 2007 Oct 12;7:66. doi: 10.1186/1472-6807-7-66 (PMC2099433; doi:10.1186/1472-6807-7-66)
Supplement: Additional file 2 — Concentration-response curves of all mutants towards NHDC. [file 1472-6807-7-66-S2.pdf]

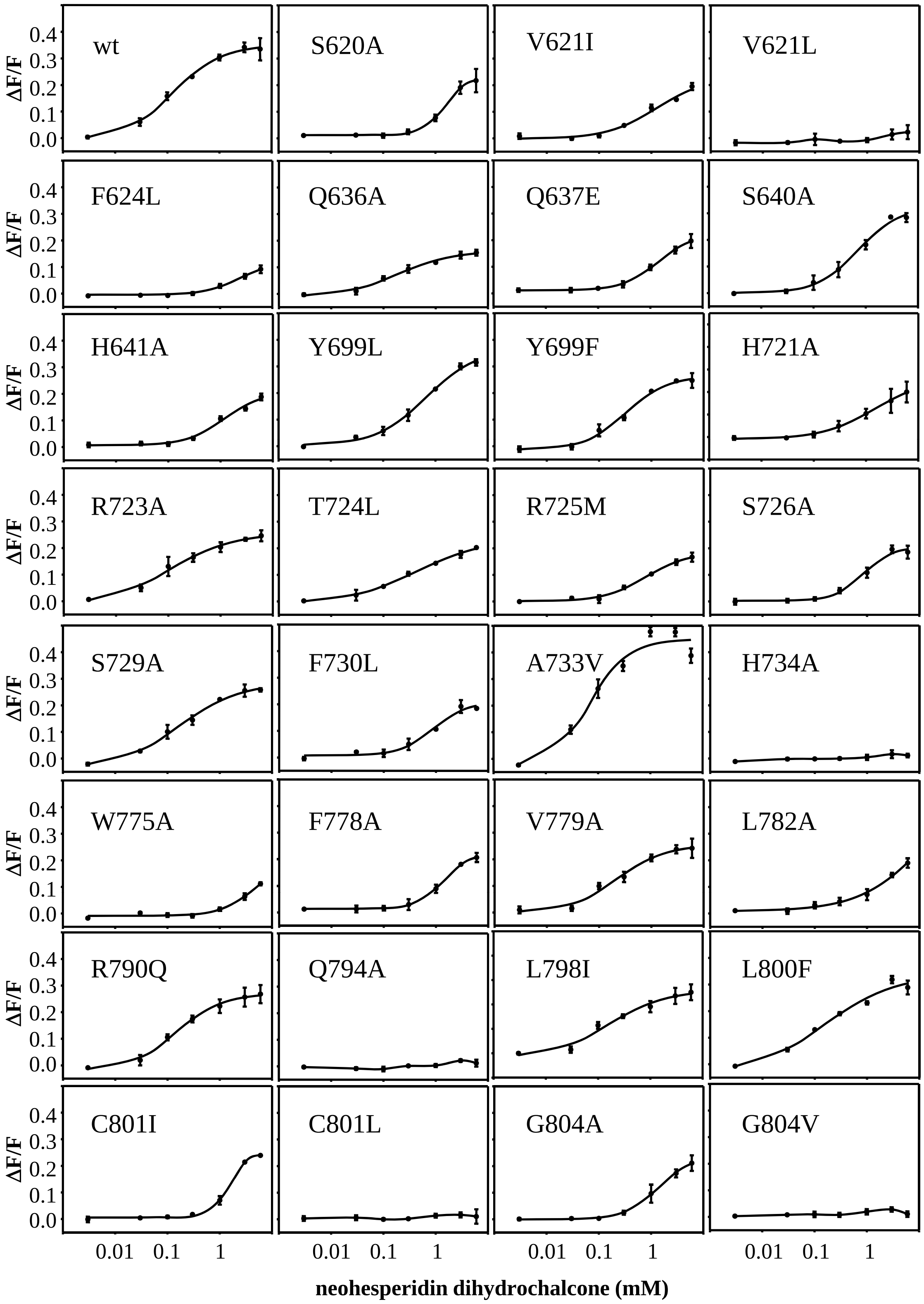

Additional figure 3. The effect of NHDC on cells expressing the hTAS1R3 mutants and hTAS1R2. HEK293T/G16Gust44 cells were transiently cotransfected with hTAS1R2 plus the indicated hTAS1R3 mutant receptors and stimulated with increasing concentrations of neohesperidin dihydrochalcone. Data are presented as the mean values plus / minus standard deviation of 3 independent transfection experiments.
